# Diagnostic Support for Selected Paediatric Pulmonary Diseases Using Answer-Pattern Recognition in Questionnaires Based on Combined Data Mining Applications—A Monocentric Observational Pilot Study

**DOI:** 10.1371/journal.pone.0135180

**Published:** 2015-08-12

**Authors:** Ann-Katrin Rother, Nicolaus Schwerk, Folke Brinkmann, Frank Klawonn, Werner Lechner, Lorenz Grigull

**Affiliations:** 1 Department of Paediatric Haematology and Oncology, University Children’s Hospital, Hanover Medical School, Hanover, Germany; 2 Department of Paediatric Pneumology, Allergy and Neonatology, University Children's Hospital, Hanover Medical School, Hanover, Germany; 3 Department of Paediatric Pneumology, University Children's Hospital, Ruhr- University Bochum, Bochum, Germany; 4 Helmholtz Centre for Infection Research, Braunschweig, Germany; 5 Improved Medical Diagnostics, Ptd. Ltd., Singapore; 6 Ostfalia University of Applied Sciences, Wolfenbuettel, Germany; University of Tübingen, GERMANY

## Abstract

**Background:**

Clinical symptoms in children with pulmonary diseases are frequently non-specific. Rare diseases such as primary ciliary dyskinesia (PCD), cystic fibrosis (CF) or protracted bacterial bronchitis (PBB) can be easily missed at the general practitioner (GP).

**Objective:**

To develop and test a questionnaire-based and data mining-supported tool providing diagnostic support for selected pulmonary diseases.

**Methods:**

First, interviews with parents of affected children were conducted and analysed. These parental observations during the pre-diagnostic time formed the basis for a new questionnaire addressing the parents’ view on the disease. Secondly, parents with a sick child (e.g. PCD, PBB) answered the questionnaire and a data base was set up. Finally, a computer program consisting of eight different classifiers (support vector machine (SVM), artificial neural network (ANN), fuzzy rule-based, random forest, logistic regression, linear discriminant analysis, naive Bayes and nearest neighbour) and an ensemble classifier was developed and trained to categorise any given new questionnaire and suggest a diagnosis. For estimating the diagnostic accuracy, we applied ten-fold stratified cross validation.

**Results:**

All questionnaires of patients suffering from CF, asthma (AS), PCD, acute bronchitis (AB) and the healthy control group were correctly diagnosed by the fusion algorithm. For the pneumonia (PM) group 19/21 (90.5%) and for the PBB group 17/18 (94.4%) correct diagnoses could be reached. The program detected the correct diagnoses with an overall sensitivity of 98.8%. Receiver operating characteristics (ROC) analyses confirmed the accuracy of this diagnostic tool. Case studies highlighted the applicability of the tool in the daily work of a GP.

**Conclusion:**

For children with symptoms of pulmonary diseases a questionnaire-based diagnostic support tool using data mining techniques exhibited good results in arriving at diagnostic suggestions. In the hands of a doctor, this tool could be of value in arousing awareness for rare pulmonary diseases such as PCD or CF.

## Introduction

Paediatricians regularly see patients with airway diseases. Recognising a rare underlying condition can be challenging because the symptoms are non-specific and often do not point towards a specific diagnosis. As a consequence, children with rare diseases might not be diagnosed at time. Various algorithms have been developed for children with cough, yet the rate of incorrect diagnoses remains high [1]. Therefore, we developed an innovative diagnostic tool to support clinicians treating children with pulmonary complaints.

In a recent cohort studied by Marchant and co-workers [2] 45% of the children referred to a tertiary clinic for evaluation of chronic cough had protracted bacterial bronchitis (PBB). It is noteworthy that many children with chronic airway infections are falsely treated for asthma resulting in side-effects [3]. Furthermore, 20–40% of children with cough receive antibiotics despite the beneficial effects of this approach remaining controversial [4–6]. Likewise, intoxication due to over-the-counter cough medications accounted for 5.7% of all emergency department visits according to US data [7], resulting in withdrawal and re-labelling of these medications [8].

This illustrates that children with chronic airway infections or an underlying rare medical condition are often at risk of incorrect diagnoses [9]: In Germany, children suffering from cystic fibrosis (CF) are often not diagnosed before the age of 5 years whilst the diagnosis is provided much earlier in countries with established neonatal screening programmes [10]. The diagnosis of primary ciliary dyskinesia (PCD) is also frequently delayed. According to Coren [11], the median age at diagnosis was 4.4 years, but very long pre-diagnostic phases have also been reported with severe consequences for patients [12]. Even conditions where diagnosis might be regarded as simple, such as asthma (AS), are regularly misdiagnosed [13].

To address this shortcoming we developed a computerised diagnostic support tool for children with airway infections using a new and straightforward questionnaire for patients’ parents. Primarily focusing on symptoms before the establishment of a diagnosis; we interviewed parents with affected children to gain insights into the parental view. Out of these observations using methods of qualitative analysis a novel questionnaire was built and completed by parents of sick children. The answers were analysed using advanced data mining techniques for pattern recognition to provide diagnostic support.

We had previously developed useful algorithms for medical diagnostic support and then generated a novel tool for the paediatric emergency department [14]. Using a combination of three data mining methods and a fusion algorithm, 18 different diagnoses (e.g. pneumonia (PM), appendicitis, meningitis) could be detected using a data set consisting of 26 clinical and laboratory parameters. By asking simple questions like exploring the medical history we now aimed at raising awareness for rare pulmonary diseases such as PCD or CF.

Here, the results of a monocentric, non-randomised pilot study for proof of concept are shown. The study evaluated the results of a questionnaire-based diagnostic tool using data mining algorithms for diagnosing children with selected pulmonary diseases (CF, PCD, AS, PBB, acute bronchitis (AB), and PM). Using the answers of patients with an already established diagnosis a data mining system was trained to recognize the different answer-patterns. With the approach of patient-centred questionnaires and data mining the diagnostic accuracy of our system reached 91–99%.

## Methods

### Parental Interviews

In this prospective monocentric pilot study we developed and evaluated a novel diagnostic support tool in children with different pulmonary diseases. First, we decided to include six frequent and rare diseases, where ‘cough and airway infection’ were one of the main symptoms, and included a control group of children without airway infection. Then interviews were performed with parents of an affected child.

Table 1 shows the cohort of patients participating in the interviews.

**Table 1:** Cohort of Patients Participating in Interviews.

Group		Age at Diagnosis	Diagnostic Latency^a^	Total Length of Interview (in minutes)
**CF**	1	1 d	0 d	44
	2	10 m	10 m	24
	3	9 y	2 y	25
**AS**	1	1 y	6 m	19
	2	3.5 y	3.5 y	25
	3	3.5 y	2 y	36
**PCD**	1	2 y	1 y	29
	2	6 y	4 y	18
	3	5.5 y	5.5 y	15
**PM**	1	7 y	7 d	8
	2	11 y	4 d	23
	3	4 y	3 d	13
**AB**	1	1.5 y	1 d	32
	2	6 m	1 d	23
**PBB**	1	3.5 y	3 y	27
	2	5 y	3 y	42

m months; y years; d days;

^a^diagnostic latency: time between 1st symptom and diagnosis of underlying disease

The focus was on the pre-diagnostic time. All parents had a child with an established diagnosis of asthma, PCD, PBB, PM, AS, AB or CF. This diagnosis was confirmed by a paediatric pulmonologist using standard diagnostic criteria. The interviewer was aware of the patient’s diagnosis. All interviews took place during a regular visit at Medical University Hospital Hanover, a tertiary university teaching hospital. After a short introduction to the topic, the parents were invited to participate in the study and gave their informed consent. To reflect differences in the individual disease presentation, we performed at least two interviews in each disease group. After completing 16 interviews this part of the data collection was ceased, as no additional categories were derived from them. It was apparent that we had achieved “theoretical saturation”, and hence, based upon the principles of qualitative research, the decision to cease was made. Theoretical saturation is based upon the assumption that after the circular sequence of data collection and analysis no additional derivative categories will be identified [15]. The Ethics Committee of the Medical University of Hanover approved the conduct of the study, and written informed consent was obtained from all participants (Approval number: 1161–2011). For children, the informed written consent was provided by the legal guardians. A narrative interview technique was chosen for optimal collection of parental observations [16, 17].

All interviews started with the same initial question (“Please tell me everything that comes into your mind regarding the development of your child, especially symptoms regarding the airways; any information you recall is important and valuable”). When the parents’ report ended, the interviewer would ask additional questions to elucidate more details from the narration. All interviews were digitally recorded and transcribed using the transcription rules of Rosenthal [17] and analysed according to the techniques of Mayring [18]. Likewise, the 16 interviews were analysed, categorised and compared. In the penultimate step, questions were formulated out of the narration, which consequently reflected both the narration and the different categories of the interviews. After exclusion of redundant questions, 335 questions remained which were then reduced to 45 final questions by three experienced paediatricians and paediatric pulmonary specialists to form the final questionnaire (S1 Fig). It was postulated that, especially for parents with a sick child, answering a questionnaire should not take longer than 10–15 minutes. This reduction to 45 questions also followed a systematic, step-wise procedure, to ensure that all observational categories, all-sub-categories, all types of observations and all different disease presentations were included. Parents then checked the final questionnaire in terms of comprehensibility. To increase the information content of the questionnaire, the answers could be scaled from 1 (no, it absolutely does not apply to my child’s symptoms) to 6 (yes, it applies completely to my child’s symptoms).

### Collection of Answered Questionnaires

After completing the questionnaire it was necessary to build up a set of data. Therefore, all parents of children with an established diagnosis of AS, PBB, PM, AB, CF or PCD who attended the hospital between January 2013 and March 2013 for a regular visit received a paper version of the questionnaire and were invited to answer the questions anonymously. Additionally, PCD patients and their parents were contacted via patient support organizations. Children without pulmonary disease were included as a control group. To facilitate replies to the questionnaire, we also created a web-based platform.

### Data Mining Techniques

In terms of data mining, proposing a diagnosis based on questionnaires corresponds to a classification problem. The target attribute is the diagnosis and the attributes used for the prediction are the answers to the questions which are given on an ordinal scale. Most classifiers are designed to handle either numerical or categorical attributes. Therefore, the ordinal scale was interpreted as a numerical scale.

Classifiers are based on different assumptions on how the classes–in our cases the diagnoses–can be identified or separated. For instance, linear discriminant analysis is based on the assumption that each class is represented by a multivariate normal distribution whereas a decision tree assumes that the classes can be separated by axes-parallel hyper-planes. None of these assumptions really fits to the questionnaire data set. Therefore, not a single classifier was chosen but an ensemble of classifiers.

Classifier ensembles [19], i.e. combinations of different classification algorithms, often lead to better predictions. The idea of applying classifier ensembles in the context of support for medical diagnosis has been described previously by the authors [14]. In the current study, however, we used a combination of eight distinctly different classifiers (SVM, ANN, fuzzy rule-based, random forest, logistic regression, linear discriminant analysis, naive Bayes and nearest neighbour) to enhance the accuracy of the diagnostic suggestion.

For a patient showing specific symptoms with respect to one of the seven diagnoses a majority of the eight classifiers gain an identical result. But for most of the patients a fusion algorithm is necessary to perform a weighted majority voting. Each classifier delivers a disease number and a corresponding probability value for each assumed diagnosis as well. The maximum total sum of all probability values for each single diagnosis points to the diagnosis with the highest relative probability. Summing up the probabilities of all classifiers for each diagnosis yields a score for each diagnosis. The diagnosis with the highest score is chosen as the proposed diagnosis but only if it exceeds a certain limit. At the end this fusion algorithm selects the most probable diagnosis in cases of different diagnostic suggestions of single classifiers and additionally was even partly able to exceed the maximal performance of the best classifier.

The evaluation of the classifier ensemble was based on 10-fold cross-validation and in addition on two additional case studies with patients who entered the hospital without knowing the final diagnoses.

## Results

### Interviews

16 interviews were conducted with parents of children with an established diagnosis. Likewise, 403 minutes of pre-diagnostic experience was collected. Using predefined steps, a questionnaire was then created reflecting parental observations.

This questionnaire contained 45 questions. The questions ranged from ‘Does your child snore?’ to ‘Would you say that your child is a rather bad eater?’ or addressed the general respiratory symptoms (‘Would you agree that your child is frequently missing school (or pre-school) due to respiratory problems?’).

In total, 170 parents of a child with a confirmed diagnosis answered the questionnaire. The majority of participants who answered the questionnaire were contacted by random during the hospital stay or during visits in the outpatient department. Here, the return rate of the questionnaire was 100%.

Only parents of children with PCD were contacted via the telephone and through the patient organization. In the second step, the questionnaire was dispatched. In total 30, questionnaires were sent out to PCD patients and we received 20 questionnaires back. In addition to that, we asked 4 parents with PCD to answer the questionnaire during the hospital stay of their child. (CF: 33 questionnaires; AS: 27; PCD: 24; PM: 21; AB: 23; PBB: 18; children without cough: 24).

Each question increased the diagnostic reliability of the questionnaire (data not shown), but selected questions provided the highest information gain for arriving at the correct diagnosis (Table 2). The complete questionnaire is available from the online material (S1 Fig).

**Table 2:** List of Questions with Highest Information Gain for the Computer.

Number in Questionnaire	Question
**11**	Did your child already suffer from pneumonia and stayed alert and happy throughout?
**21**	Did your child experience fever while suffering from pulmonary or respiratory problems?
**36**	Were you under the impression that your child is or was slower in its development than other children (e.g. learning to walk)?
**37**	Are there relatives with chronic pulmonary diseases (e.g. cystic fibrosis or similar)?
**43**	Do you perceive a whistling/wheezing sound when your child is breathing?
**44**	Do you or did you notice alternating days when your child was drowsy on one and feeling fine on the other?

### Diagnostic Accuracy in the Training Data Set

#### Cross Validation

Cross-validation is one possible approach for estimating the performance of a model on unseen data [20]. In this study, for training and validation purposes of the classifiers, stratified 10-fold cross-validation was applied to the complete data set of all 170 questionnaires of patients with a known diagnosis (including the 24 children without cough). Each part of the 10 validation data collections contained 17 data records. The classifier group was then trained on the basis of the remaining 153 records. This procedure was repeated 10- times for different subsets of 17 patients selected out of the 170 data set. Each subset of 17 patients contained roughly the same distribution of diagnoses as the whole set of 170 patients (stratified sampling).

#### Receiver operating characteristics (ROC) and Sensitivity Analysis of the Diagnostic Tool

**Fig 1:**
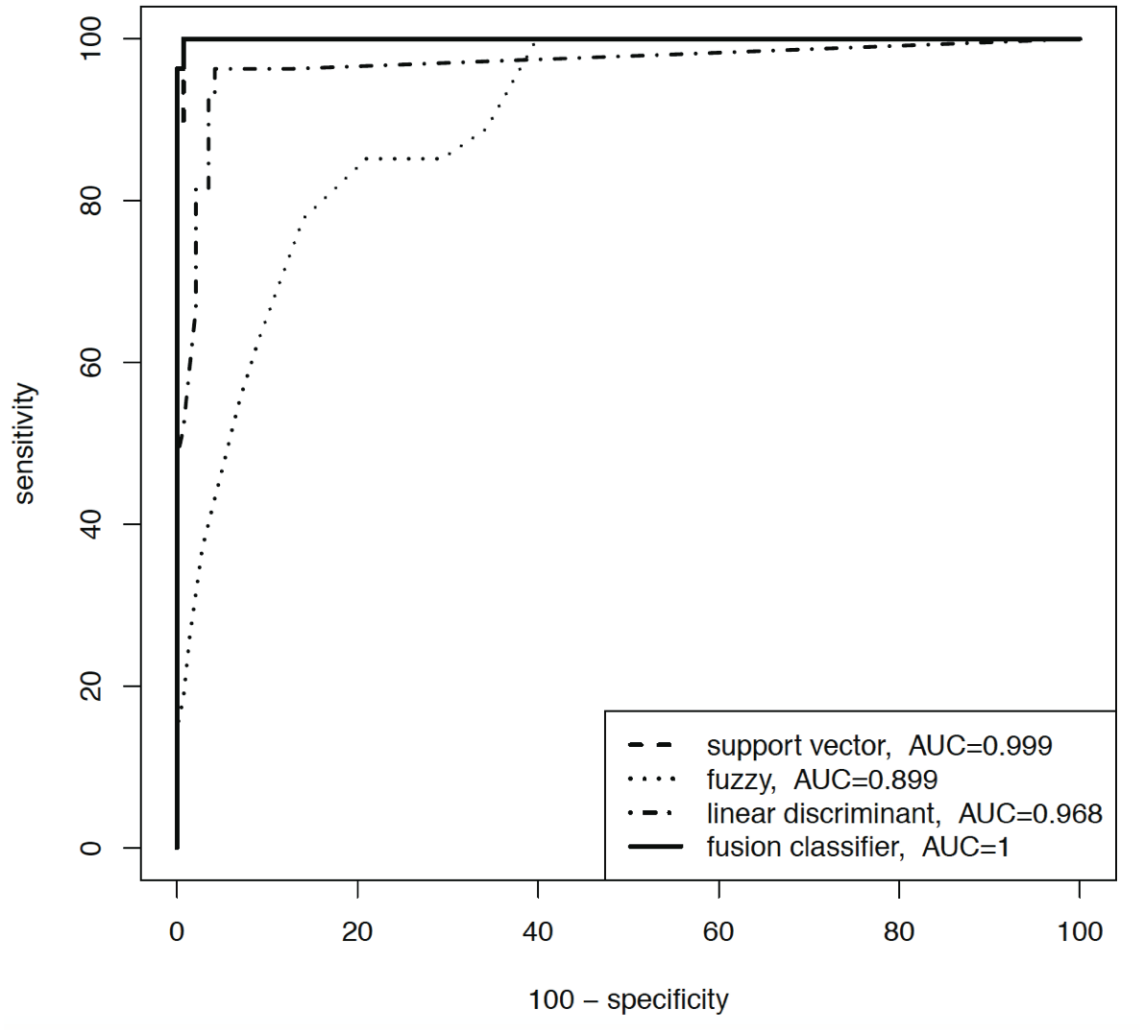
ROC curves for patients with AS. Area under the curve (AUC).

**Fig 2:**
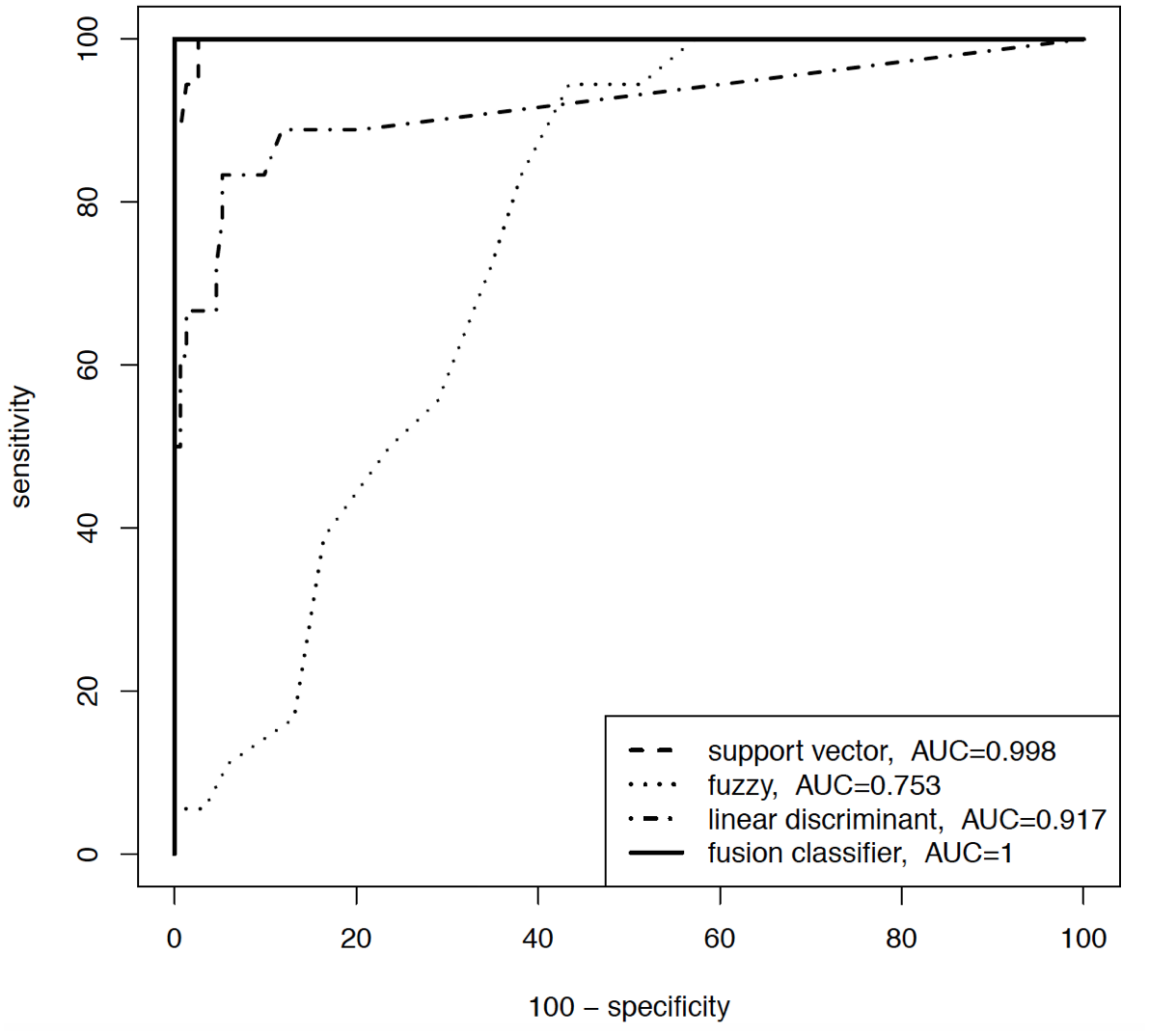
ROC curves for patients with PBB. Area under the curve (AUC).

A sensitivity analysis was applied to the data set of all study patients. Fig 1 and Fig 2 display ROC curve graphics with the associated area under the curve (AUC) for two out of seven diagnoses (AS and PBB).

Each of the single diagnoses was analysed with respect to the remaining six of the total amount of seven diagnoses. Only the performance of three classifiers (SVM, fuzzy logic and linear discriminant analysis) and the fusion algorithm is shown–although eight classifiers and their fusion were used–to enhance the understanding of the figure. The key function of the fusion algorithm is very effective, because each out of the three data mining stand-alone algorithms had certain misclassifications, but the combination in a fusion algorithm achieved the best diagnostic results (Figs 1 and 2, solid line).

In our study, all patients suffering from CF, AS, PCD and AB and the healthy control group were correctly recognised by the fusion algorithm. For the PM group, 19/21 (90.5%) and for the PBB group, 17/18 (94.4%) correct diagnoses were reached. In total, 168/170 (98.8%) patients received a correct diagnosis by the computer system.

**Fig 3:**
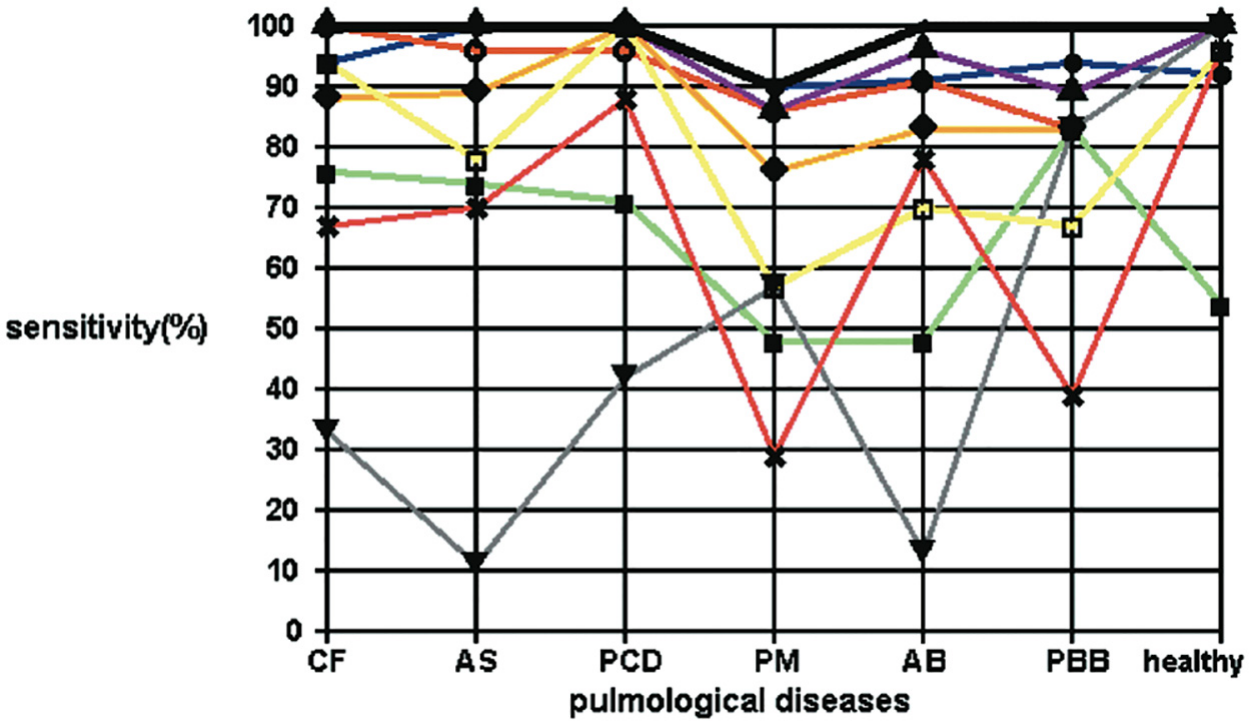
Sensitivity plots for eight classifiers and the fusion algorithm for the six selected diagnoses and the healthy control group. SVM (95%); Fuzzy (65%); ANN (94%); Random Forest (96%); Linear Regression (89%); Naive Bayes (46%); Linear Discriminant (82%); Nearest Neighbour (68%); Fusion (98%).

The coloured lines in Fig 3 indicate different sensitivities of classifying systems in different disease groups. The strength of combining classifiers becomes obvious, because the fusion algorithm (solid line) exhibits the best result for each disease. Fig 3 also demonstrates that the fusion algorithm has the potential to improve even the result of the single classifier. In the cases of AB and PBB it performs best compared to the any single stand-alone classifiers.

The fuzzy classifier showed relatively poor results as a stand-alone system. But eliminating this component would nevertheless lead to reduced overall accuracy of the results of the fusion algorithms as this classifier contributes to the final results.

#### Heat Map Analysis of Questions

**Fig 4:**
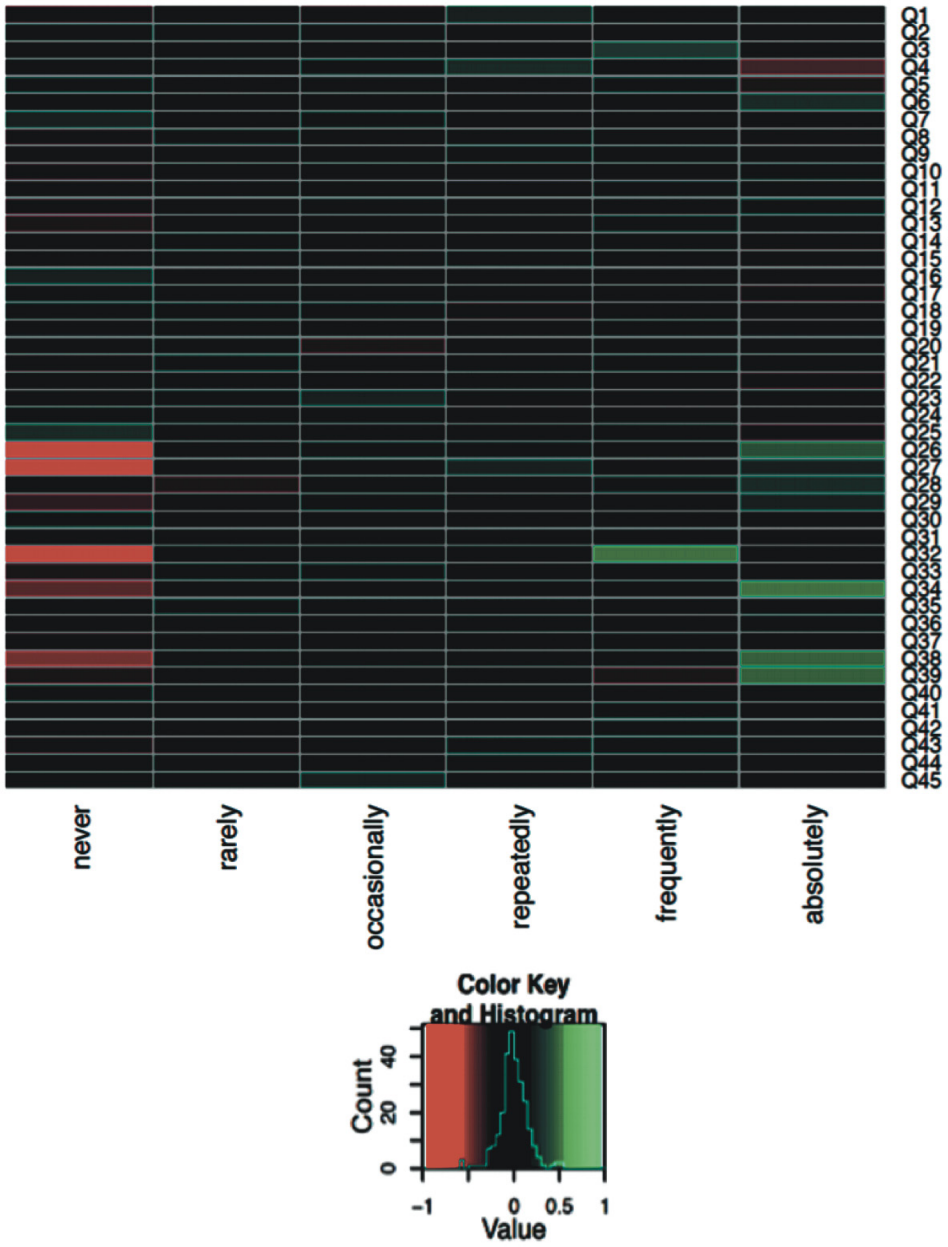
Heat map analysis of the PCD and PBB group for all 45 questions (Q1–Q45). The comparison of answer patterns between PCD and PBB parents showed that the following three questions were particularly helpful in distinguishing the two diseases. no. 26: ‘Would you say that your child is suffering from cough since birth?’ no. 34: ‘Would you say that your child’s running nose is not influenced by the season?’ no. 38: ‘Would you say that infections in your child never clear up completely, but affect the ears first and then continue by affecting the lungs?’.

**Fig 5:**
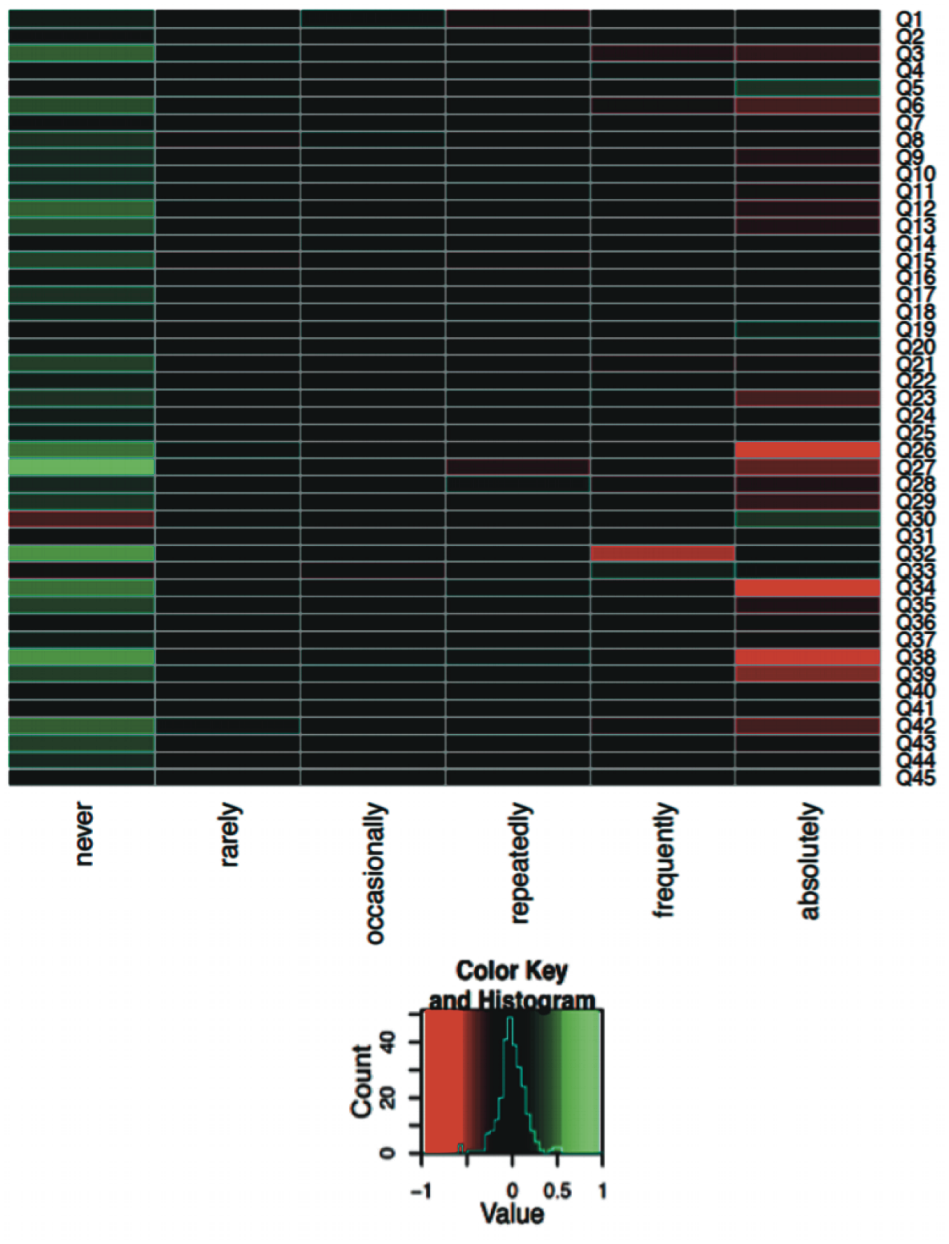
Heat map analysis of the CF and PCD group for all 45 questions (Q1–Q45). The questions no. 27: ‘Do you think that your child often suffers from an inflammation of the middle ear?’, no. 38: ‘Do you think infections in your child never clear up completely, but affect the ears first and then continue by affecting the lungs?’ were the most helpful questions for distinguishing between CF and PCD in the heat map analysis.

**Fig 6:**
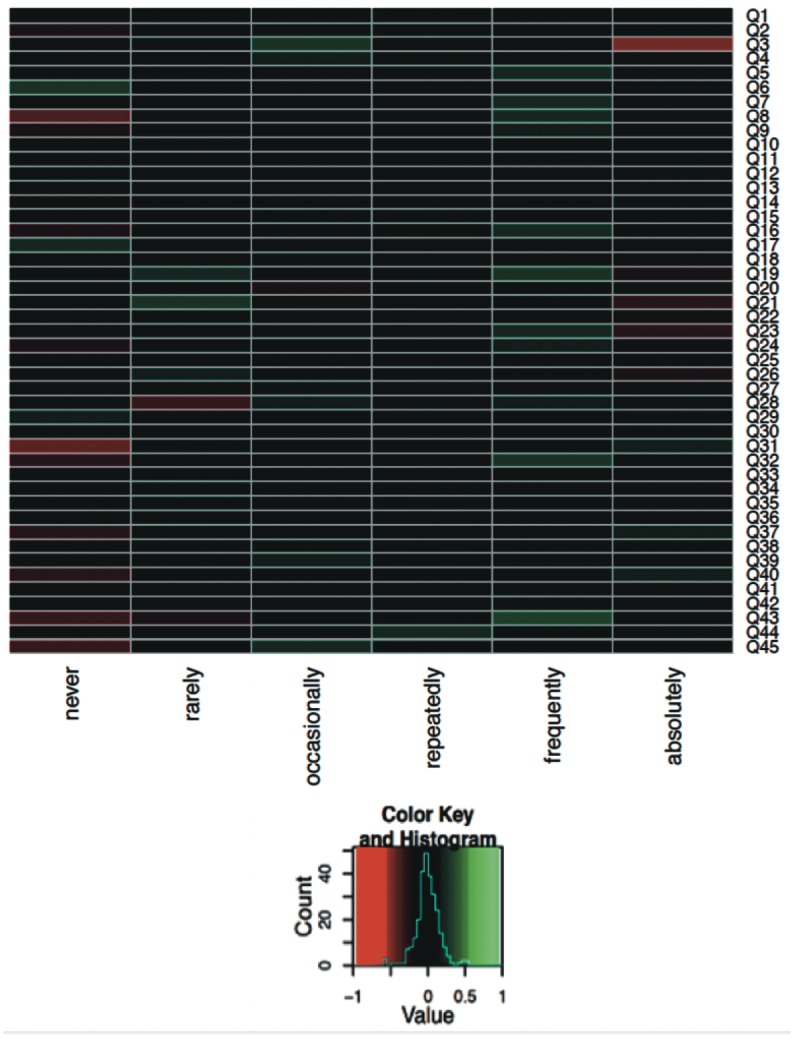
Heat map analysis of the AS and PBB group for all 45 questions (Q1–Q45). The heat map analysis of this pair indicates that one question was more supportive in differentiating between AS and PBB patients than the others: no. 31: ‘Are there any allergies detected in your child?’.

To further understand the links between the answer patterns in the different disease groups a heat map was generated for each pair of diagnoses (Figs 4–6; only selected pairs are shown). For each question and each possible answer the difference of the relative frequencies for selected pairs of diagnoses was computed. The largest possible value of 1 for a specific question and a specific answer means that all parents from the first diagnosis group had chosen this answer whereas no parents from the other diagnosis group had selected this answer. The smallest possible value of -1 means the opposite, i.e. no parents from the first diagnosis group had chosen the corresponding answer but all parents from the second diagnosis group had selected this answer. In both cases, this would offer a hint that the corresponding question has a high potential to distinguish between the two diagnoses. The value 0 means that the relative frequency of the specific answer is equal for both diagnoses. If this is the case for all possible answers for a specific question, this indicates that this question alone cannot contribute to distinguishing between the two diagnoses. Heat map analyses in diseases where differential diagnosis is extremely challenging are displayed and discussed (Figs 4–6).

Heat map analysis of the answer pattern between PBB and AS illustrates the inherent problem of medical diagnosis. Where the clinical phenotype is quite similar, the answer pattern looks similar as well (Fig 6). However, the combined data mining algorithms used in this study were nevertheless able to distinguish the answer patterns with satisfactory reliability. A heat map comparing all possible diagnostic pairs which illustrates the most important questions for distinction is available as S2 Fig.

### Case studies

Case study 1: A 6 year old girl was admitted to our hospital for further evaluation of a chronic cough. On admission, she was clinically well, afebrile, tcSaO2 98%, respiratory rate 18/min. On auscultation wet rales on the right side were detected. She had been unwell since 2 months of age, suffering from recurrent airway infections each winter. Due to recurrent middle-ear infections, she had undergone an adenotomy and she received t-tubes but without improvement. Sweat test analysis was normal, and asthma therapy was ineffective. A chest x-ray 3 months prior to hospitalisation showed an infiltrate, so that oral antibiotic treatment was initiated. The girl had no known allergies, non-consanguineous parents and a healthy 8-year old sister. During hospitalisation, a diagnostic bronchoscopy, a CT scan of the chest and ciliary function testing were performed. The results confirmed the diagnosis of PBB and she was commenced on a 6 week antibiotic treatment with an inhalation regime. Both parents answered the questionnaire, the computer diagnosis was: PBB: 49%; PCD 43%.

Case study 2: A 6 year old boy, the first born of healthy non-consanguineous parents, had a background history including early neonatal PM, which had required one day of artificial ventilation. He subsequently had been discharged home well after a 4 week hospitalisation. During his second year of life he was commenced on inhalation therapy (salbutamol, corticosteroid) by his general paediatrician for recurrent episodes of cough and wheeze. After his third birthday, his family consulted a university hospital paediatric pulmonologist for a second opinion. Post negative immunological and sweat tests, his diagnosis was given as AS with obstructive bronchitis. A possible differential diagnosis of PCD was suggested, but diagnostic tests, including normal dynein arms, were inconclusive. PCD was not reconsidered by another paediatric pulmonologist until three years later, with more than 8 episodes of recurrent PM. Repeated PCD diagnostic testing at that time demonstrated PCD.

Both parents answered the questionnaire during a regular visit at our hospital shortly after the diagnosis was confirmed. The computer diagnosis was quite clear, rendering PCD the most possible diagnosis (46%), followed by PBB (33%). Of note, the main medical diagnosis of AS was not considered by the computer.

## Discussion

In this pilot study for proof of concept we could show that combining past medical history with modern mathematics resulted in helpful diagnostic suggestions for children with broncho-pulmonary symptoms. Cough is one of the most frequent reasons for a medical consultation [21]. In patients with chronic cough, a broad range of differential diagnoses must be taken into consideration [2]. Obviously, coming to the right diagnosis efficiently is a major challenge for physicians. Numerous diagnoses require different diagnostic algorithms and clinical pathways are scarce [22]. Subsequently we aimed at developing a pilot tool for supporting clinicians to distinguish six different pulmonary diseases in children by answering a questionnaire during the waiting time.

Today there is often significant diagnostic delay, missed diagnoses, incorrect treatments and unnecessary tests for patients with chronic cough [2]: For instance, patients with PCD can have delays of up to 14 years from onset of first symptom until diagnosis [23]. Therefore we aimed at utilizing pattern recognition with data mining methods to help direct the doctor’s attention for even rare paediatric pulmonary diseases. Of note, the diagnosis of PCD relies on sophisticated examination of the ultrastructure and function of the cilia [24]. However, before such an investigation will be initiated, one must first suspect PCD. With regard to diagnostic delay, especially in children with PCD, earlier diagnosis might result in an improvement in the long-term outcome [25]. Focussing on children with chronic airway infections and addressing possible drawbacks of existing clinical decision support systems (CDSS), a questionnaire-based solution focussing on parental observations seemed logical and was systematically developed. In this pilot study, the computer recognised correctly the answering pattern in 90.5–99% of the children with CF, AS, PCD, PM, AB and PBB.

In children with chronic wet cough, PBB is increasingly recognized as an important differential diagnosis [26]. Certainly, the diagnosis of PBB relies on several diagnostic criteria: (1) wet cough for ≥ 4 weeks, (2) “absence of specific pointers to indicate an alternative specific cause of cough“, and (3) response to antibiotics (amoxicillin/clavulanate) with resolution of cough within 2 weeks [2, 27, 28]. The questionnaire based tool presented here could help raise awareness of PBB as an important differential diagnosis in children with prolonged (wet) cough.

In patients with wheezing, cough and recurrent dyspnoea, asthma is a likely diagnosis, but both over-diagnosis and underestimation frequently occurs [29]. Data from Luks and colleagues and Linden-Smith demonstrated an incorrect asthma diagnosis in 30 to 41% of cases. As a consequence, 71% of incorrectly diagnosed ‘asthma-patients’ receive asthma medication resulting in potentially significant side-effects and additional costs [13]. Consequently, an additional and easy-to use questionnaire might prove beneficial for parents and physicians to avoid these consequences.

Not surprisingly, our results confirm and underline the power of parental observations, which has yet been only seldom used for detecting rare diseases. This information is traditionally only collected in the doctor–patient/parent dialogue. Given the positive results documented, it is somewhat surprising that computerised decision support is not more widely used. This may be due to unsuccessful work in this field in the seventies and eighties, when so-called expert systems were generated for medical diagnosis [30]. Despite many efforts, these attempts have proved ineffective, mainly because medicine is rarely based on simple ‘yes’ or ‘no’ decisions. However, modern data mining methods now provide the capacity for powerful data exploration far beyond simple expert systems because, rather than merely imitating human reasoning, they exploit statistical data patterns [19, 31, 32].

While various algorithms for the work-up in children with chronic cough exist, the extrapolation of adult-based strategies into a paediatric population can be difficult [2]. Sometimes the reason for the cough might be obvious, but especially children with rare diseases frequently receive wrong diagnoses and medications because an underlying condition for the chronic airway infection was overlooked [9–12, 33]. Therefore, diagnostic support, which is independent of radiological or even clinical input, but driven solely by the strength of parental observation and data mining techniques, might be a useful diagnostic support tool. Accordingly, this study and the diagnostic tool under investigation might fill a gap, because many studies suggest that diagnostic support is needed for children with chronic cough or for patients with rare pulmonary diseases where diagnostic latency may result in undesired long-term sequaelae [9, 10, 25].

Unlike earlier computer-aided diagnostic support methods dating back to the 1970s, when scientists unsuccessfully aimed to enhance diagnostic accuracy and reduce error by using databases and mathematical algorithms [30, 34], data mining technologies today are proven to be powerful tools for medical decision support with good accuracy in selected diagnostic areas [35– 37].

The results of this study are new and innovative for several reasons; first, supporting clinical diagnosis successfully using data mining applications is not yet established. Second, combining the process of ‘history taking’ using questions derived from interviews with data mining techniques is like combining ‘old-school medicine’ (i.e. history taking) with the twenty-first-century use of mathematical methods, as is common practice in commercial companies such as Google, Amazon, etc..

Using a combination of eight different classifiers for medical diagnosis is not a completely new concept, but despite its promising results, it is still not common to apply classifier ensembles to medical diagnosis [38].

To avoid any additional data entries–traditionally disliked by physicians–we developed a CDSS tool that is completed by patients (or their relatives) in the waiting area (e.g. using a tablet PC), but the resulting diagnostic suggestion (which is generated immediately) is only displayed to the physician. Consequently, this diagnostic support tool does not add to doctors' workload while still providing valuable diagnostic support with good accuracy.

One might argue that answering the questionnaire may interfere with the workflow of a consultation. Yet, the mean waiting time at the paediatrician in Germany is about 29 minutes. Hence answering a questionnaire during this time should not hinder the visit [39].

Beyond that, we decided to include patients' (or parents') perception to arrive at a diagnosis. Using techniques from the field of qualitative research we analysed the parents’ point of view especially in the pre-diagnostic phase. The questionnaire was pre-tested and carefully re-read by parents with different cultural background. Today, the questionnaire is available in German, English and Chinese language. Whether or not the sensitivity will be different in other cultures will be evaluated in future studies. These perceptions were finally merged into a questionnaire encompassing 45 questions resulting in useful diagnostic suggestions. As the time required for answering a questionnaire is a disadvantage of this type of diagnostic support tool-we limited the number of incorporated questions. Further reduction of the questions, however, reduced the diagnostic accuracy of the tool (data not shown).

Parental understanding plays an important role in diagnosis and disease management as reported for AS patients in several studies [40]. In addition, misunderstanding between doctors and parents regarding signs and symptoms are frequent, as reported in detail for the asthmatic child [41, 42]. This prevalent problem was simply bypassed in our study by integrating patients’ language and perception.

Our study has several limitations, however. First, we conducted interviews and collected questionnaires only within a limited, but heterogeneous population. Nevertheless and remarkably enough, data from qualitative research indicate that already a limited number of interviews reflects the themes/categories of the whole cohort [43]. Several other studies successfully used even smaller cohorts for interviews, but also reached the level of theoretical saturation [44]

In the group of PM patients especially, we sampled younger and older children as well as typical and atypical PM. In addition, the number of answered questionnaires in this group was low, thus resulting in insufficient output findings (90.5% correct diagnoses). Second, only a selected number of diseases were incorporated in the data set as it was designed to prove the concept and different socio-cultural backgrounds have not been considered. This might lead to misleading diagnostic suggestions. Just like a young doctor, who only encounters a very limited range of diseases, our diagnostic tool currently features a set of only seven differentials. Nevertheless, unlike other diagnostic support tools, data mining methods are self-learning and expandable. Therefore, inclusion of any new or additional diagnoses (e.g. gastro-oesophageal reflux disease (GERD) or lung malformation) would be simple and has already been demonstrated in a previous project [14]. Case-studies of children suffering from rare pulmonary diseases illustrated the applicability of the diagnostic support tool.

A possible diagnosis of PCD in case study 2 might help direct a physician to the problem, but will definitely not eliminate all the tests required for definitive diagnosis. The expected advantage might be in avoiding unnecessary investigations and helping with targeted history taking. Whether or not a questionnaire-based tool will assist to decrease costs was not analysed in this study and should be addressed in future studies.

Our data carries the inherent limitations of a monocentric study. Recent work of our group with questionnaire-based diagnostic in the field of neuromuscular diseases (Grigull et al., Muscle & Nerve, under submission / unpublished data) gives support for the hypothesis that the diagnostic tool under investigation here will reach a slightly lower diagnostic accuracy in a prospective trial, which is currently planned for the questionnaire under discussion.

The need for diagnostic support in paediatric PM or AB patients is debatable. Clinical diagnosis of PM without chest X-ray results in over-diagnosis [45]. Data from Denmark illustrate high usage of antibiotics in airway infections of viral origin [33]. Our data demonstrated good results in diagnosing PM and AB from questionnaires, which could be beneficial for the clinical diagnosis of pneumonia patients.

In rare diseases such as PCD, CF and PBB, current data support the notion that diagnostic delay in these patients promotes severe complications such as bronchiectasis [12, 46, 47]. A recent survey published by Boon and co-workers underlines the clinical variability and diagnostic delay in patients with PCD [48].

In our study, very good results were found for diagnosing PCD only by analysing the answering pattern of 45 questions and a data mining supported analysis of the responses. Although the diagnosis of PCD will always have to rely on additional investigations, patient and doctor should benefit from an earlier decision to confirm the presumption. Data mining has, to our knowledge, not yet been used in such a context, but its usability for diagnostic support has frequently been underscored [31, 32, 36]. The power of pattern recognition especially, which is obvious in questionnaires, is a core strength of statistical learning.

Our diagnostic tool in the hands of the GP caring for a child with recurrent pulmonary infections or chronic airway infections might not prompt an immediate diagnosis, but should shorten the diagnostic latency by provoking additional tests for patients to rule out an underlying (possibly) condition and might shorten the diagnostic latency in rare diseases.

Questionnaire-based and data mining supported diagnosis have proved to work well in children with selected pulmonary diseases. Modern mathematical procedures were able to distinguish different response patterns in a large amount of data. Surprisingly good results for the detection of PCD patients gives room for hope that diagnostic delay will be shortened even in rare paediatric pulmonary diseases. The value of parents’ observation during the pre-diagnostic time is highlighted by our data. Certainly, the ultimate diagnosis should remain in the hands of the doctor and additional investigations, but an easily manageable support tool might prove beneficial.

## Supporting Information

S1 Fig Original German questionnaire used in the study. (PDF)

S2 Fig Heat map comparing all possible diagnostic pairs. All possible diagnostic pairs are illustrated. The most important questions for distinction are green dots.
(PDF)

S1 Table IMD raw dataset. (XLSX)

S1 Text English translation of the questionnaire. (PDF)
