# Supplementary material for: Diagnostic Support for Selected Paediatric Pulmonary Diseases Using Answer-Pattern Recognition in Questionnaires Based on Combined Data Mining Applications—A Monocentric Observational Pilot Study
Source: PLoS One. 2015 Aug 12;10(8):e0135180. doi: 10.1371/journal.pone.0135180 (PMC4534438; doi:10.1371/journal.pone.0135180)
Supplement: S1 Fig — (PDF) [file pone.0135180.s001.pdf]

Eltern wissen mehr!

- Welche Beobachtungen haben Sie gemacht? -

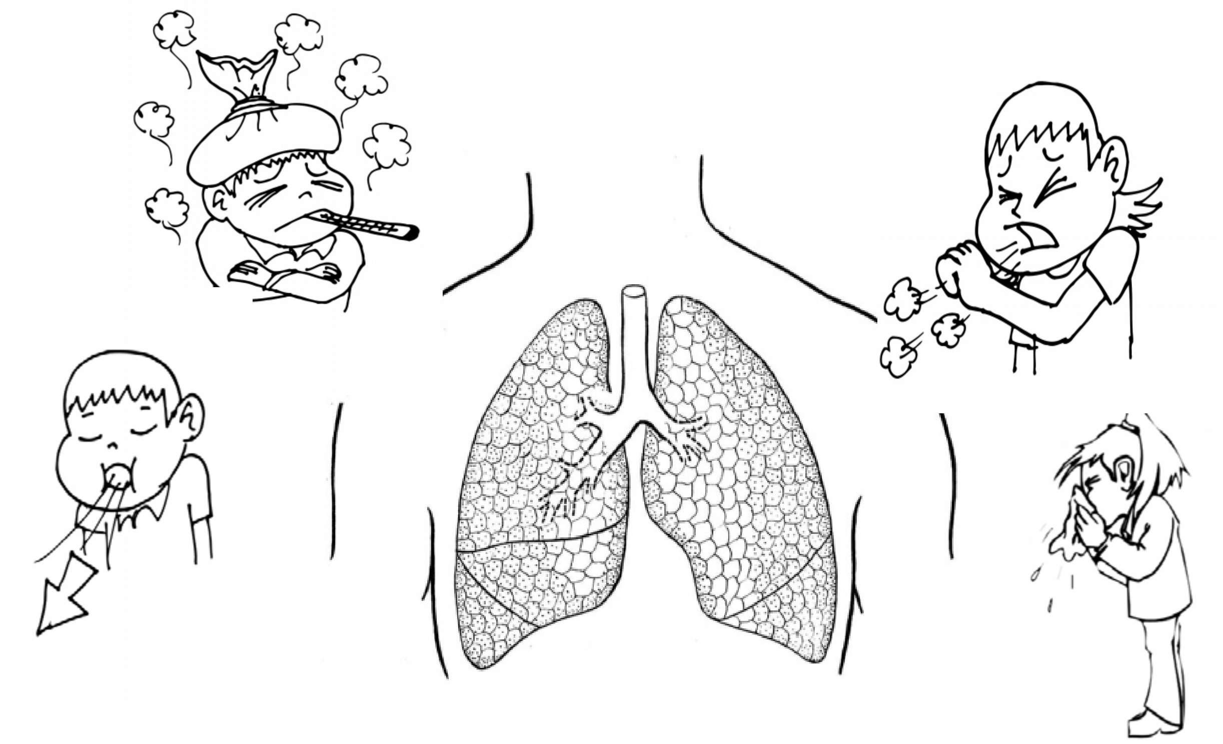

Eine Umfrage der Medizinische Hochschule Hannover **zum Pilotprojekt:**

DaMiPu

Fragebogenbasierte und Data-Mining gestützte Diagnostik ausgewählter pulmonaler Erkrankungen bei Kindern

(2012)

Medizinische Hochschule Hannover
Carl-Neuberg-Straße 1
30625 Hannover

Ihre Ansprechpartner:
PD Dr. Lorenz Grigull Tel. 0511-532-3221
Ann-Katrin Rother Tel. 0170/2839719
Dr. Nicolaus Schwerk Tel. 0511-532-3221
Dr. Folke Brinkmann Tel. 0511-532-3221

Abteilungen für Päd. Pneumonologie sowie Päd. Hämatologie und Onkologie

DaMiPu: Fragebogenbasierte und Data-Mining gestützte Diagnostik ausgewählter pulmonaler Erkrankungen bei Kindern (2012)

Liebe Eltern, Hannover, im Juni 2012

wir arbeiten an einem Projekt, mit Hilfe von Fragebögen die Diagnostik von ausgewählten Lungenerkrankungen bei Kindern zu unterstützen. Hierzu haben wir im vergangenen Jahr Interviews mit Eltern von Kindern mit verschiedenen Lungenerkrankungen durchgeführt, denn wir wollen so das Erfahrungswissen der Eltern besser nutzbar machen. Daraus ist der beiliegende Fragenkatalog entstanden. Nun brauchen wir ***Ihre Hilfe*** und ***Ihr Erfahrungswissen.***

Wir möchten Sie höflich bitten, sich ein paar Minuten für das Ausfüllen des Fragebogens zu nehmen. Bitte erinnern Sie sich hierzu an die Zeit, **bevor** die Beschwerden Ihres Kindes einen Namen (eine „Diagnose“) hatten. Kreuzen Sie einfach auf dem Fragebogen an, was *am besten* auf Ihr Kind in der Zeit **vor der Diagnose zutraf**. Hat Ihr Kind momentan das erste Mal Beschwerden, beziehen Sie sich bitte auf die letzen Tage und Wochen. Unser Ziel ist es, mit Hilfe Ihrer Antworten die Diagnostik zukünftig unterstützen zu können. Alle Angaben sind natürlich ***komplett anonymisiert*** und werden nur für das geschilderte Projekt verwendet. Bitte vergessen Sie nicht, Alter und Diagnose Ihres Kindes anzugeben. Sprechen Sie uns gerne bei Fragen oder Rückmeldungen an.

Vielen Dank für Ihre Mithilfe!

PD Dr. L. Grigull Dr. N. Schwerk Dr. F. Brinkmann cand. med. A.-K. Rother

(Kontakt: Dr. L. Grigull; Email: grigull.lorenz@mh-hannover; Fon: 0511-532-3221)

Fragebogen "DaMiPu"

Ausfüllhinweise: Bitte verwenden Sie einen blauen oder schwarzen Kugelschreiber oder einen Bleistift !
Markieren Sie so: ☐☒☐☐☐ falsch: ✓☐
Korrigieren Sie so: ☐■☐☒☐

Dieser Fragebogen wird maschinell erfasst. Bitte überschreiben Sie nicht die aufgedruckten Nummern oder die Eckmarkierungen auf den Seiten. Anmerkungen außerhalb der Anmerkungsfelder werden nicht erfasst.

Im Folgenden finden Sie eine Reihe von Fragen. Bitte antworten Sie, je nachdem ob Sie persönlich meinen, dass eine Feststellung eher "völlig zutrifft" oder eher "überhaupt nicht zutrifft" mit Angaben zwischen 1 (= trifft überhaupt nicht zu) und 6 (= trifft völlig zu). Sie können dabei gar nichts falsch machen, denn es gibt keine für jede Person zutreffenden Antworten. Antworten Sie bitte aufrichtig und ohne lange zu überlegen; lassen Sie bitte keine Antworten aus. Die Untersuchung dient allein wissenschaftlichen Forschungszwecken. Der Datenschutz ist gewährleistet; geben Sie bitte ihren Namen nicht an, sondern machen Sie bitte lediglich die Angaben zum betroffenen Kind!

Geschlecht Ihres Kindes: ☐ Junge ☐ Mädchen

Alter Ihres Kindes heute? ☐ Jahre

Alter Ihres Kindes bei Auftreten der ersten Symptome? ☐ Jahre

Alter Ihres Kindes bei Stellung der Diagnose? ☐ Jahre

Welche Diagnose wurde bei Ihrem Kind gestellt? ☐

	trifft überhaupt nicht zu 1	2	3	4	5	6 trifft völlig zu
1) **Schnarcht** Ihr Kind?	☐	☐	☐	☐	☐	☐
2) Leidet Ihr Kind manchmal unter **Luftnot**?	☐	☐	☐	☐	☐	☐
3) Kann man sagen, dass Ihr Kind **stark verschleimt** ist?	☐	☐	☐	☐	☐	☐
4) Hat bzw. hatte Ihr Kind einen **trockenen** Husten?	☐	☐	☐	☐	☐	☐
5) Gibt es auch Zeiten ohne Atembeschwerden bei Ihrem Kind?	☐	☐	☐	☐	☐	☐
6) Haben Sie das Gefühl, Sie bekommen Ihr Kind nicht mehr **hustenfrei**?	☐	☐	☐	☐	☐	☐
7) Besteht bzw. bestand während einer Erkältung auch **verstärkt Luftnot**?	☐	☐	☐	☐	☐	☐
8) Trifft es zu, dass Ihrem Kind schneller als früher **"die Puste" ausgeht** (bei Aktivitäten, die es normalerweise stundenlang ausüben kann oder konnte, z.B. Spielen auf dem Spielplatz, Toben mit Freunden, Sportunterricht)?	☐	☐	☐	☐	☐	☐
9) Tritt bzw. trat **Husten** bevorzugt nach großer Anstrengung auf (z. B. Sport, wildes Toben, lange Fahrradtour)?	☐	☐	☐	☐	☐	☐
10) Hatte Ihr Kind bereits eine **Lungenentzündung**?	☐	☐	☐	☐	☐	☐
11) Hatte Ihr Kind bereits eine Lungenentzündung und war während dessen trotzdem munter und fröhlich?	☐	☐	☐	☐	☐	☐
12) Hatte Ihr Kind bereits eine Lungenentzündung, und nach dem Absetzen des Antibiotikums **folgte gleich die nächste Lungenentzündung**?	☐	☐	☐	☐	☐	☐
13) War Ihr Kind wegen Problemen mit der Lunge bereits im ersten Lebensjahr **häufiger krank**?	☐	☐	☐	☐	☐	☐
14) Leidet oder litt ihr Kind unter **Neurodermitis** (krankhaftes Hautjucken)?	☐	☐	☐	☐	☐	☐
15) Schläft bzw. schlief Ihr Kind schlecht, weil es ständig husten muss/musste?	☐	☐	☐	☐	☐	☐
16) Kündigen sich Anfälle von Atemnot mit einer **"käsigen oder komischen" Gesichtsfarbe** an?	☐	☐	☐	☐	☐	☐
17) Würden Sie sagen, dass man den Husten Ihres Kindes als **festsitzend** beschreiben kann/konnte?	☐	☐	☐	☐	☐	☐

Bitte wenden!

	trifft überhaupt nicht zu 1	2	3	4	5	6 trifft völlig zu
18) Finden oder fanden Sie ihr Kind auffallend **schlapp und/oder abgeschlagen (müde)**?	☐	☐	☐	☐	☐	☐
19) Geht bzw. ging auftretende **Luftnot**, auch ohne Medikamente, nach **einer kurzen Erholungsphase** von alleine wieder weg?	☐	☐	☐	☐	☐	☐
20) Würden Sie sagen, dass Ihr Kind ein eher **schlechter Esser** ist?	☐	☐	☐	☐	☐	☐
21) Hat bzw. hatte Ihr Kind bei Lungen- oder **Atemwegsproblemen** auch **Fieber**?	☐	☐	☐	☐	☐	☐
22) Hatte Ihr Kind zunächst **Schnupfen** und im weiteren Verlauf kam **Husten** dazu?	☐	☐	☐	☐	☐	☐
23) Würden Sie ihr Kind als **infektanfällig** bezeichnen?	☐	☐	☐	☐	☐	☐
24) Kommt bzw. kam es bei **Wetterumschwüngen** zu Atemnotanfällen?	☐	☐	☐	☐	☐	☐
25) Tritt oder trat der Husten Ihres Kindes **anfallsartig** auf?	☐	☐	☐	☐	☐	☐
26) Hustet Ihr Kind gewissermaßen **von Geburt an** durchgehend?	☐	☐	☐	☐	☐	☐
27) Finden Sie, dass Ihr Kind **öfter** unter Mittelohrentzündungen leidet bzw. litt?	☐	☐	☐	☐	☐	☐
28) **Atmet** Ihr Kind eher **durch den Mund**?	☐	☐	☐	☐	☐	☐
29) Hatte Ihr Kind sofort nach der **Geburt Luftnot**?	☐	☐	☐	☐	☐	☐
30) Bestand bereits im ersten Lebensmonat nach der Geburt **ein Darmproblem** (z.B. erhebliche Verstopfung, Operation, Darmverschluss)?	☐	☐	☐	☐	☐	☐
31) Sind bei Ihrem Kind **Allergien** festgestellt worden?	☐	☐	☐	☐	☐	☐
32) Wurden bei Ihrem Kind bereits die "**Mandeln**" und/oder "**Polypen**" entfernt?	☐	☐	☐	☐	☐	☐
33) Kann man sagen, dass Ihr Kind auffallend häufig **Durchfall** hat?	☐	☐	☐	☐	☐	☐
34) Liegt bei Ihrem Kind ein **jahreszeitenunabhängiger "Dauerschnupfen"** vor?	☐	☐	☐	☐	☐	☐
35) Finden Sie, dass Ihr Kind **im Vergleich zu Gleichaltrigen** besonders häufig **Atemprobleme** hat und vielleicht daher auch häufiger im Kindergarten/in der Schule fehlt?	☐	☐	☐	☐	☐	☐
36) Haben bzw. hatten Sie das Gefühl, Ihr Kind ist/war in der **Entwicklung seiner Bewegungen** (z. B. spätes Laufen lernen) langsamer als seine Altersgenossen?	☐	☐	☐	☐	☐	☐
37) Gibt es **Verwandte** mit einer chronischen Lungenerkrankung (z.B. Asthma, Mukoviszidose, Primäre Ciliäre Dyskinesie, Chronische Bronchitis oder ähnliches)?	☐	☐	☐	☐	☐	☐
38) Trifft es zu, dass **Infekte** bei Ihrem Kind **nicht richtig weggehen**, dass beispielsweise erst die Ohren, dann die Lungen usw. immer wieder betroffen sind?	☐	☐	☐	☐	☐	☐
39) Haben bzw. hatten Sie schon länger "irgendwie" das Gefühl, dass mit Ihrem Kind **etwas nicht stimmt**?	☐	☐	☐	☐	☐	☐
40) Bestehen bzw. bestanden während der Haupt-**Pollenflugzeit** (März bis September) Atemprobleme?	☐	☐	☐	☐	☐	☐
41) Musste Ihr Kind bereits an der **Lunge operiert** werden?	☐	☐	☐	☐	☐	☐
42) Haben/Hatten Sie den Eindruck, dass man bei Ihrem Kind beim Atmen manchmal **"rasselnde Atemgeräusche"** hört/hörte?	☐	☐	☐	☐	☐	☐
43) Hören Sie ein **Pfeifen**, wenn Ihr Kind atmet?	☐	☐	☐	☐	☐	☐
44) **Wechseln** bzw. wechselten sich Tage mit **Schlappheit** und Tage mit **gutem Befinden** ab?	☐	☐	☐	☐	☐	☐
45) Gibt bzw. gab es Tage an denen Ihr Kind sehr stark hustet/ hustete, die von Tagen **mit völliger Beschwerdefreiheit** abgelöst werden/wurden?	☐	☐	☐	☐	☐	☐

Herzlichen Dank für Ihre Zeit und die Beantwortung der Fragen!
