# Supplementary figures and images for: Diagnostic Support for Selected Paediatric Pulmonary Diseases Using Answer-Pattern Recognition in Questionnaires Based on Combined Data Mining Applications—A Monocentric Observational Pilot Study
Source: PLoS One. 2015 Aug 12;10(8):e0135180. doi: 10.1371/journal.pone.0135180 (PMC4534438; doi:10.1371/journal.pone.0135180)

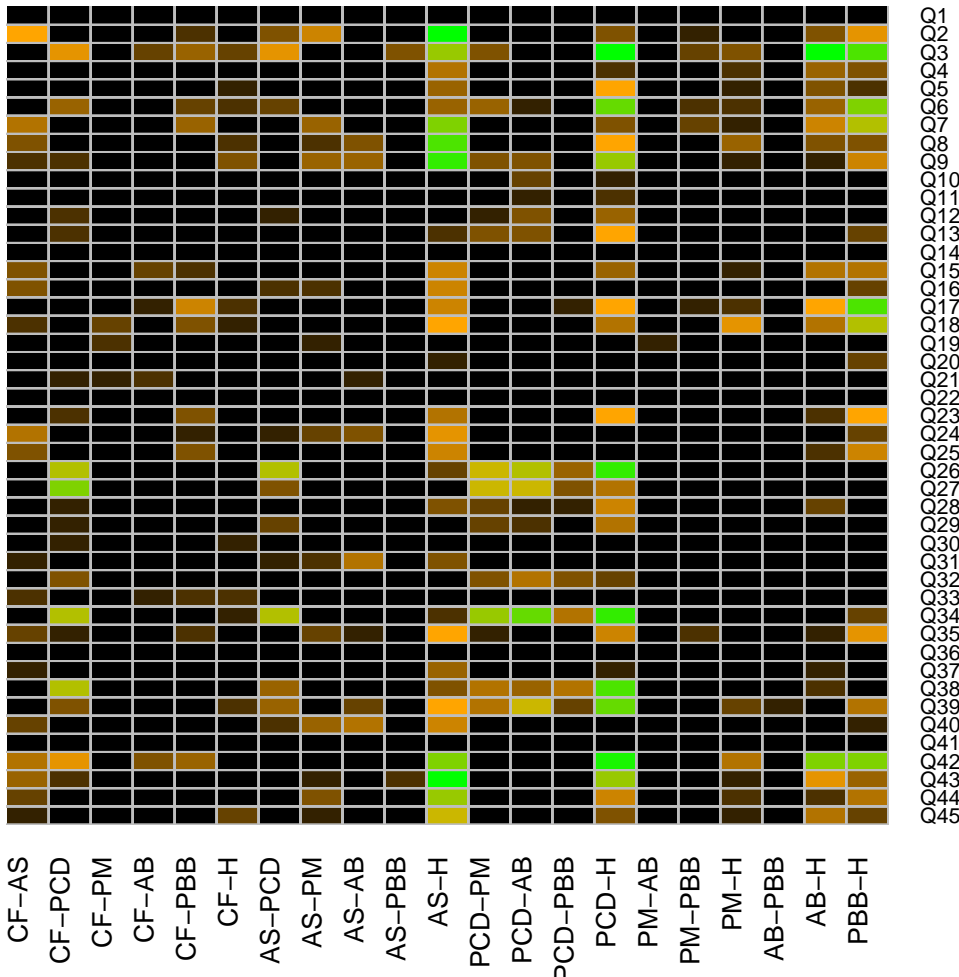

Supplement: S2 Fig — All possible diagnostic pairs are illustrated. The most important questions for distinction are green dots. (PDF) [file pone.0135180.s002.pdf]
