# Supplementary material for: Diagnostic Support for Selected Paediatric Pulmonary Diseases Using Answer-Pattern Recognition in Questionnaires Based on Combined Data Mining Applications—A Monocentric Observational Pilot Study
Source: PLoS One. 2015 Aug 12;10(8):e0135180. doi: 10.1371/journal.pone.0135180 (PMC4534438; doi:10.1371/journal.pone.0135180)
Supplement: S1 Text — (PDF) [file pone.0135180.s004.pdf]

Translated Questionnaire

How old is your child?

1. Does your child have a dry cough?

2. Does your child suffer from breathing difficulties?

3. Would you say that your child has a very slimy throat?

4. Does your child snore?

5. Does your child go without any breathing difficulties?

6. Do you have the feeling that, you are not able to get your child free of cough?

7. Exists during a cold also increased distress?

8. Would you say that your child is “out of breath” more easily, e.g. during physical activities, sports?

9. Do you experience coughing after exhausting activities such as sports?

10. Did your child already suffer a lung infection?

11. Did your child already suffer a lung infection but remained cheerful?

12. Did your child already a suffer a lung infection and experienced a subsequent lung infection after finshing an antibiotics treatment?

13. Did your child experience lung problems in its first year after birth?

14. Does your child suffer from neurodermitis?

15. Does your child suffer from sleeping difficulties due to persistent coughing?

16. Do attacks of breathing difficulties come with pale or unusual face colouring?

17. Would you describe your child's cough as deep-seated?

18. Is your child frequently tired and lacking energy?

19. Do the breathing difficulties pass after short phases of recuperation without medication?

20. Would you consider that your child has bad appetite, is eating insufficiently?

21. Does your child have fever when experiencing lung or respiratory problems?

22. Did your child suffer initially from sneezing followed by a cough?

23. Would you consider your child prone to infections?

24. Do you observe respiratory difficulties following a change of weather?

25. Does the cough your child experiences occur in bursts?

26. Does your child suffer from coughing since its birth?

27. Do you observe that your child frequently suffers from middle ear infections (otitis)?

28. Does your child breathe mainly through the mouth?

29. Did your child experience breathing difficulties right after birth?

30. Did your child experience gastro-intestinal difficulties in the first month after birth?

31. Does your child suffer from allergies?

32. Did your child undergo surgery for tonsils or polypes?

33. Does your child suffer frequently from diarrhea?

34. Does your child suffer from chronic head colds independent of seasonal changes?

35. Do you notice that your child suffers more from respiratory difficulties than its peers resulting in more times absent from pre-school or school?

36. Do you notice that your child was slower to develop its motions, e.g. learning to walk than its peers?

37. Are there cases of chronic pulmonic diseases in the immediate family?

38. Would you say that infections your child experiences never properly pass, e.g. an infection of the ears is followed by another affecting the lungs?

39. Did you already have the general feeling that somehow not all was quite right with your child’s health?

40. Do you notice respiratory difficulties during pollination times March-September)?

41. Did your child undergo lung surgery?

42. Do you notice a “rattling” sound when your child breathes?

43. Do you notice a whistling sound when your child breathes?

44. Do you notice that days of fitness alternate with days of fatigue in your child?

45. Do you notice days when your child coughs or coughs severely alternating with days with no symptoms at all?
